# Rapidly Progressive Peritoneal Carcinomatosis Originating From Mucinous Appendiceal Adenocarcinoma: A Case Report

**DOI:** 10.7759/cureus.116775

**Published:** 2026-09-23

**Authors:** Cole Verble, Kalyn Powers Verble

**Affiliations:** 1 Physical Medicine and Rehabilitation, University of Florida (UF) Health Shands Hospital, Gainesville, USA; 2 Pharmacology and Therapeutics, University of Florida (UF) Health Shands Hospital, Gainesville, USA

**Keywords:** carcinomatosis peritoneal, crs/hipec, cytoreductive surgery (crs), hyperthermic intraperitoneal chemotherapy, mucinous appendiceal adenocarcinoma, pseudomyxoma peritonei

## Abstract

Malignant neoplasms of the appendix are rare. These neoplasms can be further delineated into either colonic or cystic types. A cystic type appendiceal tumor is often referred to as an appendiceal mucocele due to its production of jelly-like mucus. Mucinous appendiceal adenocarcinoma (MAA) is a malignant appendiceal mucocele associated with nonspecific symptoms and often found incidentally when other abdominal work-up is performed. If the appendix ruptures, the peritoneal cavity and organs are coated with mucus-producing cells, leading to the complication pseudomyxoma peritonei (PMP). If found late in the disease progression, it can further develop into peritoneal carcinomatosis, which is typically treated with cytoreductive surgery (CRS) and heated intraperitoneal chemotherapy (HIPEC).

A 28-year-old male with no past medical history arrived at the emergency department for longstanding constipation. Upon workup, he was found to have stage IV MAA with dissemination into the peritoneal cavity leading to PMP and peritoneal carcinomatosis. The mucin in his abdominal cavity had proliferated and solidified to the point where CRS/HIPEC was unable to be performed. He received multiple rounds of palliative chemotherapy; however, it failed to shrink any tumors or lessen mucin burden in order to receive CRS/HIPEC.

This case demonstrates the importance of early detection of MAA. It also illustrates that even in healthy young adults, advanced peritoneal carcinomatosis may impede CRS/HIPEC from being performed. In the current literature, most patients with peritoneal carcinomatosis, unless contraindicated, are able to receive CRS/HIPEC.

## Introduction

Malignant neoplasms of the appendix are exceedingly rare, with an age-adjusted incidence of roughly 0.12 per one million people [1]. Primary adenocarcinomas make up only around 20% of malignant neoplasms of the appendix [2]. Primary adenocarcinomas of the appendix can be further broken down into two subcategories: cystic or colonic. The colonic type is aptly named due to its resemblance to typical colorectal cancer on a biological and histological level. The cystic type, often referred to as “mucinous”, has no histological resemblance to colorectal cancer and has differing pathological characteristics [3]. Another important distinction between mucinous appendiceal adenocarcinoma (MAA) and other forms of colorectal or appendiceal cancers is that there are no established risk factors for the development of this rare form of cancer [4]. This may be, in part, due to its extreme rarity and lack of present-day clinical research. 

The prognosis of MAA largely depends on the timing of when the tumor or tumors are discovered. Many cases of MAA are incidentally discovered when the patient is having an unrelated abdominal surgery or when the tumor(s) are mistaken for acute appendicitis [5,6]. For diagnosed appendiceal adenocarcinomas, it is typically recommended for patients to undergo a right hemicolectomy to prevent the development of pseudomyxoma peritonei (PMP) or peritoneal carcinomatosis prior to other forms of therapy [7,8]. The current standard for treatment of peritoneal dissemination is cytoreductive surgery (CRS), where a surgeon physically removes the buildup of mucin within the peritoneal cavity, followed by a heated intraperitoneal chemotherapy (HIPEC) bath to prevent further mucinous production [7,8]. There are currently no reported case studies of rapidly progressive MAA where CRS/HIPEC was not performed due to hardening of mucin. In the article, we present the following case in accordance with the CARE reporting checklist of a 28-year-old who suffered from a rapidly progressive form of MAA that was too far advanced to perform CRS/HIPEC.

## Case presentation

The patient was a 28-year-old Caucasian male with no past medical history who presented to the emergency department in September 2024 due to lack of a bowel movement for over a week. He had taken multiple doses of magnesium citrate and was only able to pass flatulence. On physical exam, the patient had mild abdominal distention and minimal abdominal pain to palpation in the left and right lower quadrants with no rebound tenderness. The initial abdominal/pelvis computed tomography (CT) scan seen in Figure 1 and Figure 2 showed a mass on the appendix measuring 6x5x3cm that was concerning for a mucinous neoplasm. The CT also showed appendiceal wall thickening and multiple fecaliths. The patient has no family history of Crohn’s disease, ulcerative colitis, or intestinal cancers. The patient underwent a laparoscopic appendectomy where a large appendiceal neoplasm was located and removed for cytological examination. A right hemicolectomy was not performed given noticeable metastasis to the right lower quadrant peritoneum, pelvis, and multiple areas on the small bowel. Given these findings, the patient was diagnosed with stage IV MAA. CRS/HIPEC was not considered at this time to allow for post-operative recovery from recent appendectomy and pending final pathology results. The pathology report noted G3 invasive mucinous adenocarcinoma with HER2 equivocal positivity, KRAS G12D, SMAD4 L529Yfs*8, TP53 R273C, and PTEN deletion positivity. The patient was sent home and initiated on his first cycle of outpatient chemotherapy with leucovorin, fluorouracil, and oxaliplatin (FOLFOX) with the aim of shrinking tumor burden. 

**Figure 1:**
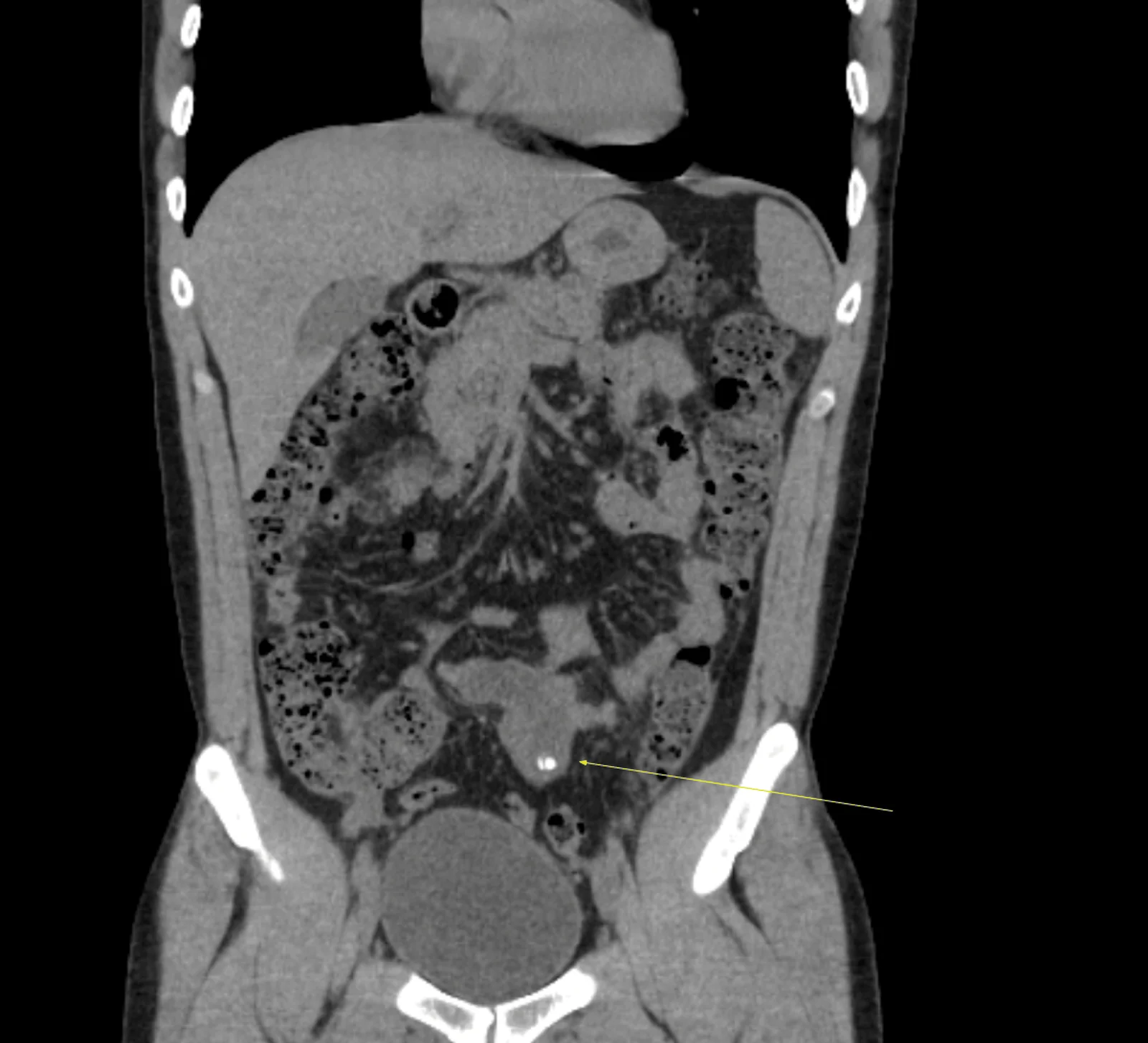
CT scan of the abdomen/pelvis without contrast in coronal view showing an enlarged appendix measuring 6x5x3 cm in size in the middle lower quadrant.

**Figure 2:**
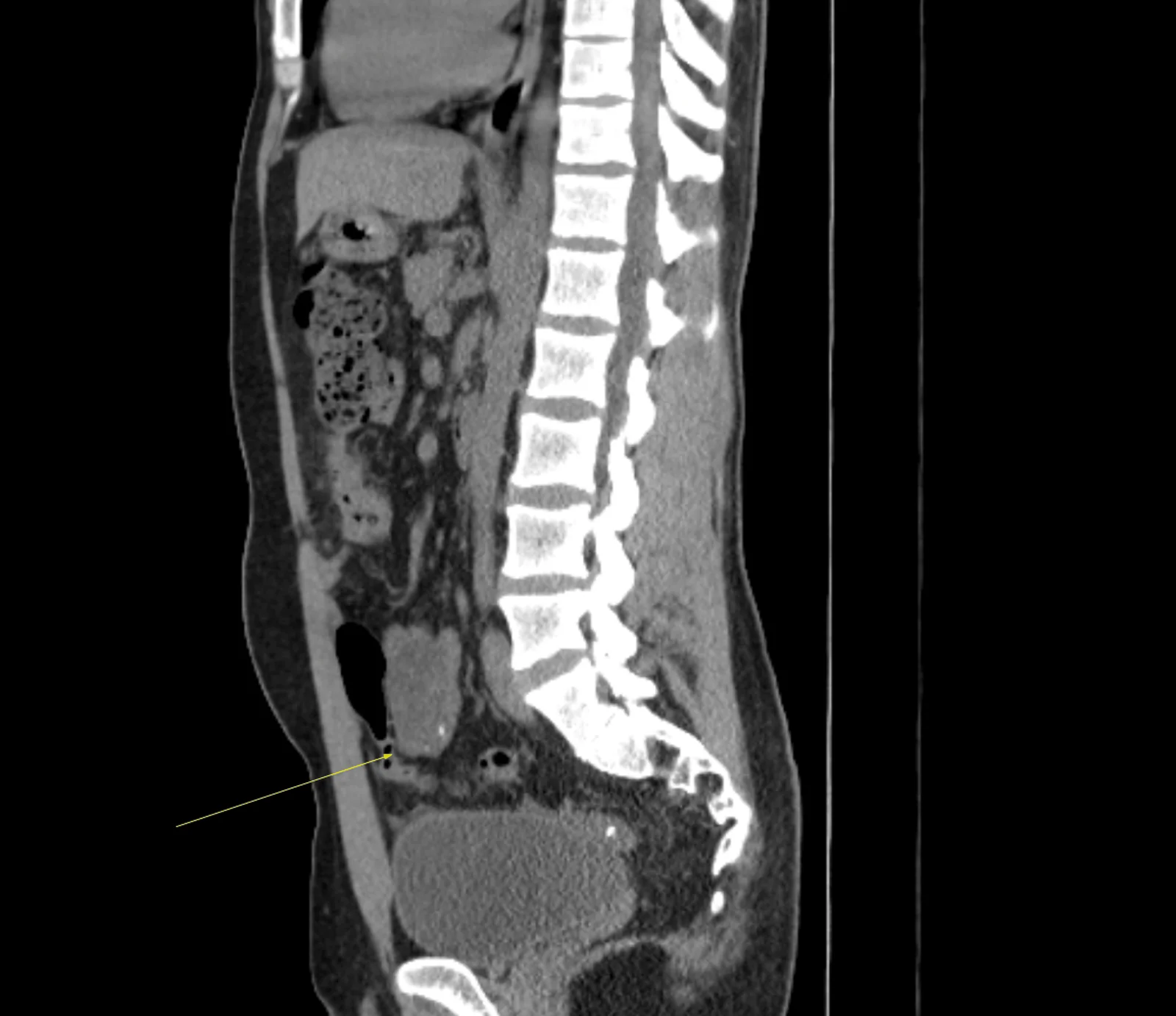
CT scan of the abdomen/pelvis without contrast in sagittal view showing an enlarged appendix measuring 6x5x3 cm in size.

In November 2024, the patient returned to the emergency department with an eight-day history of absent bowel movements, severe abdominal pain, abdominal distention, nausea, and vomiting. The repeat CT scan of the abdomen/pelvis showed large-volume malignant ascites and bowel obstruction. After a five-liter therapeutic paracentesis in the left lower quadrant, a colonoscopy was performed in which the gastroenterologist was unable to pass the scope through an unknown area of the bowel. The decision was made to have an exploratory laparotomy, which eventually led to a hemicolectomy with diverting colostomy due to the large tumor burden precipitating bowel obstruction. At this time, he was diagnosed with peritoneal carcinomatosis. The patient was initiated on a second cycle of chemotherapy with FOLFOX in December 2024 and placed on total parenteral nutrition. 

In January 2025, another diagnostic laparotomy was performed due to persistent nausea and vomiting relieved only by a nasogastric tube set to suction. The colorectal surgeon was able to place a venting intra-abdominal tube during the surgery to alleviate abdominal pressure, noting extensive solidified mucin production. At this time, the patient’s tumor burden and mucin buildup were too extensive to perform CRS/HIPEC. After a thorough discussion with the patient, the patient’s family, oncology, and palliative care about overall concern for prognosis, the decision was made to initiate his third cycle of FOLFOX in hopes of prolonging his survival to see the birth of his second child in March 2025. He was transferred to a facility closer to his home where he completed his chemotherapy, and he eventually succumbed to his ailments in April 2025, eight months after initial diagnosis. A concise and detailed timeline illustrating the patient's medical course clearly can be observed in Figure 3.

**Figure 3:**
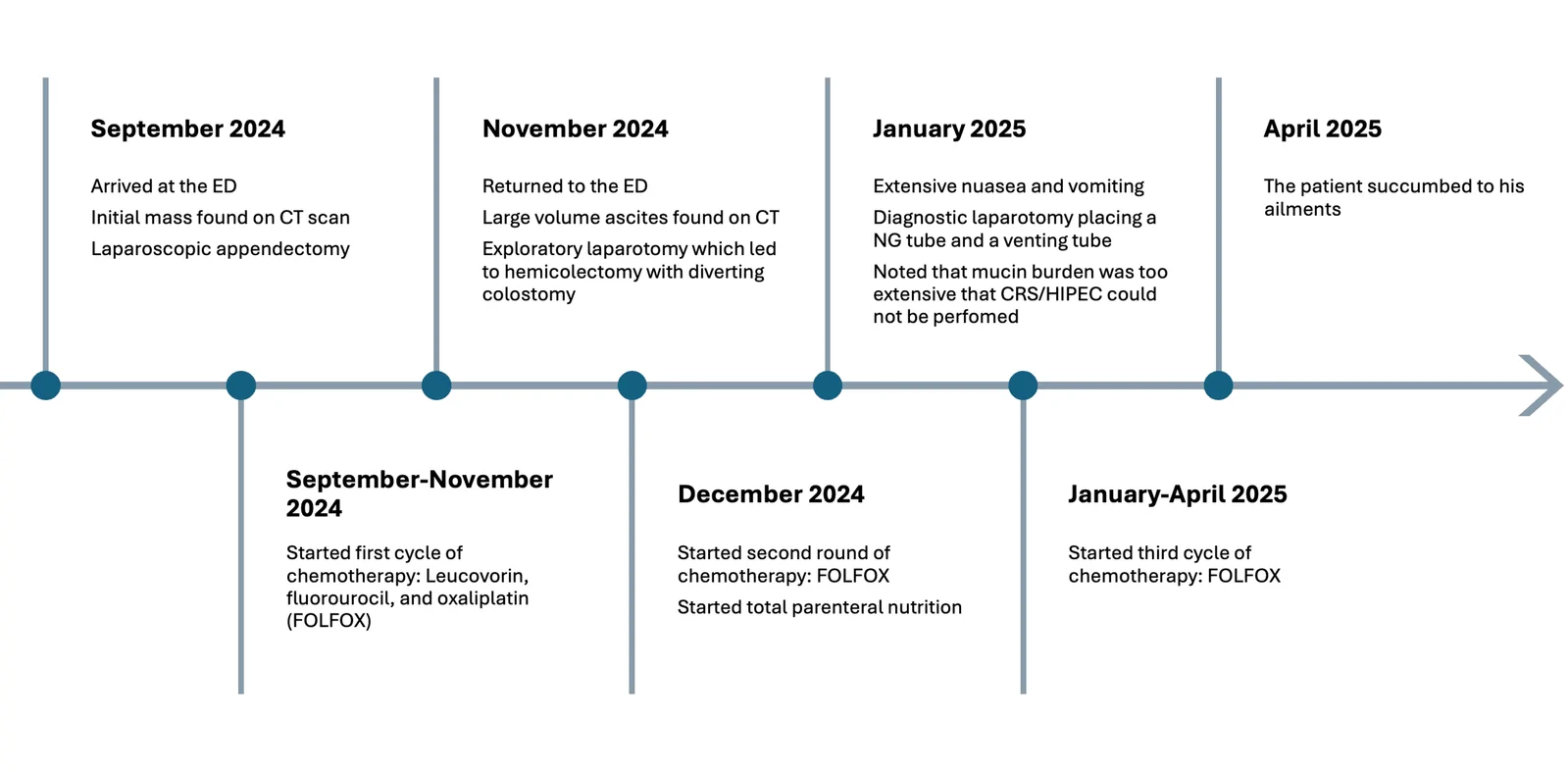
Patient's clinical course timeline. CRS: Cytoreductive surgery; HIPEC: heated intraperitoneal chemotherapy; NG: nasogastric

## Discussion

Primary appendiceal adenocarcinoma is exceedingly rare and makes up less than 0.5% of all gastrointestinal malignancies. Of the two types, cystic type (MAA) is characterized by the overproduction of mucin, a glycoprotein produced by epithelial cells with a jelly-like appearance. Since primary appendiceal adenocarcinoma is often discovered incidentally, the average age of onset for MAA is 60 years old and it typically has no risk factors and a nonspecific symptom profile such as abdominal discomfort, constipation, bloating, and nausea [4]. The patient in this case being 28 years old with advanced peritoneal disease at the time of diagnosis illustrates a rare and unfortunate discovery. 

The metastasis of MAA usually develops after appendix rupture, leading to leakage of mucin-producing cells into the peritoneal cavity. This results in PMP, a condition not unique to MAA and more commonly arising from mucinous tumors originating from the ovary, colonic, or biliary tracts [9]. PMP is characterized by vague symptoms of abdominal pain, with markedly persistent abdominal distention and constipation indicative of poorer prognosis. PMP is often diagnosed incidentally alongside MAA during exploratory laparoscopy and can be present in varying degrees. The diagnosis of PMP is indicative of peritoneal dissemination of mucinous cells, which may lead to diagnosis of peritoneal carcinomatosis, a metastasis of cancer cells to the lining of the abdominal cavity [4]. Severe complications of PMP include mucinous ascites and frozen abdomen, both of which the patient in this case experienced. Mucinous ascites are indicative of disease progression and illustrate the spread of malignant, fluid-producing cells in the abdominal cavity. 

In addition to this patient's age, the extent of the disease at the time of discovery was uncommon as well. The clinical course of MAA appears to be influenced by staging and histological features at diagnosis. Since MAA is primarily discovered incidentally during surgical evaluation of another condition, it may be common to see a variety of disease severity and progression at initial diagnosis. However, in the current literature, most appendiceal adenocarcinomas are low-grade and rarely have peritoneal spread or lymphatic involvement [9]. In this case, the patient's initial CT scan noted abdominal involvement, indicating the appendiceal cells had likely already metastasized into the abdominal cavity. During the appendectomy, the surgeon noted peritoneal spread in the right and left lower quadrants, pelvis, and omentum with minimal production of mucin. Although tumor sizes were not formally documented, the majority of biopsies demonstrated margins >0.5 cm. Using this information, a retrospective peritoneal cancer index (PCI) score was calculated to estimate the extent of peritoneal spread on initial diagnosis. The documented biopsy margins suggest that he had a score of at least 2 in most of the affected areas, thus leading to an estimated PCI score of at least 18 on initial diagnosis [10]. Although the patient's initial estimated PCI score was relatively moderate at the time of diagnosis, it was determined to be poorly differentiated and a high-grade G3 metastasis, leading to a poor prognosis. In addition, he also had lymphovascular involvement. Individuals who were diagnosed with stage IV MAA had a significantly lower five-year survival rate compared to individuals with stages I-III [2]. According to Xie et al., their nomogram would give this patient a score of 285.5, which would roughly give a 0.67 for a one-year survival rate (around eight months) [2]. The day of initial symptoms and diagnosis to the day of the patient's death was approximately eight months. This case demonstrates that the spread of the tumor cells into the peritoneal cavity happened prior to any invasive procedures into the abdominal cavity. Invasive procedures with entry into the abdominal cavity outside the midline via the linea alba may have an increased risk of seeding the abdominal wall with cancerous cells [11]. This patient underwent multiple procedures that disrupted the abdominal wall outside of the midline. While a causal relationship cannot be established, these additional procedures may have also contributed to his rapid disease progression.

In the current literature, the colonic-type adenocarcinoma has been reported to have a poorer prognosis than MAA; however, it is hypothesized that the mucin produced by MAA may act as a physical barrier to treatment options, which may increase the difficulty for the utilization of specific treatments like CRS/HIPEC in advanced cases [7,8]. Despite the progression of the disease, the majority of patients with MAA can receive CRS and HIPEC [12]. In the study by Leiritz et al., they had 21 patients diagnosed with MAA, 11 of whom had peritoneal metastasis [12]. All 11 patients with peritoneal metastasis received CRS and HIPEC, and eight of the remaining 10 patients received prophylactic CRS and HIPEC. The remaining two patients were unable to receive CRS and HIPEC due to other medical reasons [12].

Based on the current literature, it is imperative to perform CRS and HIPEC in individuals who have peritoneal metastasis. There are currently no clinical guidelines in place for individuals with PMP who have a significant mucinous burden in the peritoneum. The decision was made in this case to perform the hemicolectomy with diverting colostomy and try chemotherapy initially in the hope that the mucin and tumor burden would diminish while the patient healed from the hemicolectomy. Other than salvage chemotherapy that may potentially reduce the mucin burden, there are currently no Food and Drug Administration (FDA) approved medications that can act as mucolytics for MAA. Since there are no alternative medications to reduce or slow mucin production, CRS/HIPEC is essential for the treatment of PMP. Even though CRS/HIPEC is reported as one of the best treatments for PMP, there are still concerns regarding morbidity and mortality [9]. Although the effectiveness of systemic chemotherapy may be influenced by the specific histology and tumor grade, patients with stage IV MAA did not have much benefit from systemic chemotherapy [2].

To summarize this patient’s initial presentation, he had a relatively low mucin burden on initial diagnosis, and the surgeon did not have issues breaching the abdominal cavity for appendectomy. As per current recommendations, a hemicolectomy was performed before significant mucin accumulation while surgeons were still able to access the abdominal cavity. His initial PCI score was likely around 18, which did not preclude him from receiving CRS/HIPEC. Within two months, he progressed with other symptoms associated with poor prognosis, metastatic ascites, and continued nausea to the point of malnourishment. Once he was considered healed from his later hemicolectomy, the plan was to pursue CRS/HIPEC treatment; however, the mucin had hardened and thickened to a degree that exploring the abdominal cavity was no longer possible. Salvage chemotherapy with FOLFOX did not have an effect, and he expired around eight months after his initial diagnosis. 

## Conclusions

The purpose of this case report is to add to the current lack of literature with regard to young persons diagnosed with Stage IV MAA and PMP. This case may help distinguish criteria for possible early intervention in cases with younger and healthier individuals. This may also allow more research into the role that genetics plays in progression and the aggressive nature of MAA. This report is consistent with the current life-expectancy results cited, given current treatment options. 
